# Connecting Methods to Policy: A Narrative Review of Approaches for Conducting Policy‐Relevant Alcohol Research

**DOI:** 10.1111/acer.70438

**Published:** 2026-11-05

**Authors:** Christina F. Mair, Joseph Alejandro, Christopher N. Morrison, Toben F. Nelson

**Affiliations:** ^1^ Department of Behavioral and Community Health Sciences University of Pittsburgh School of Public Health USA; ^2^ Center for Social Dynamics and Community Health University of Pittsburgh School of Public Health USA; ^3^ Department of Chronic Disease Epidemiology Yale School of Public Health USA; ^4^ Department of Epidemiology and Preventive Medicine, School of Public Health and Preventive Medicine Monash University USA; ^5^ Division of Epidemiology and Community Health, School of Public Health University of Minnesota USA

**Keywords:** alcohol policy, artificial intelligence, narrative review, spatial analysis, system dynamics modeling

## Abstract

Alcohol consumption imposes a substantial public health burden in the United States, yet public and political support for alcohol control policies remains limited despite a strong evidence base. This stems partly from methodological challenges, including difficulties measuring policy implementation and enforcement, accounting for local variation, and assessing long‐term effects, and partly from gaps in translating findings into actionable insights for policymakers and the public. In this narrative review, we highlight three methodological approaches that can strengthen policy‐relevant alcohol research: system dynamics modeling, spatial analysis, and artificial intelligence (AI) tools for policy surveillance. Each is illustrated through a policy example (minimum unit pricing, regulating alcohol outlet density, and limiting hours of sale) to demonstrate how these methods can generate actionable insights for policymakers and the public. We conclude with recommendations for conducting policy‐relevant, methodologically rigorous research: codeveloping research questions with stakeholders, grounding research in specific theories of change, accounting for policy restrictiveness and enforcement, aligning methods with research questions, and planning for dissemination from the outset. Together, these strategies can help bridge the gap between evidence and action, ultimately strengthening support for effective alcohol control policies.

## Graphical abstract

Barriers to effective alcohol policy research include the steady relaxation of alcohol control policies and reduced enforcement at the local level; methodological challenges in measurement, accounting for local variation, and evaluating long‐term effects; and a research‐practice disconnect. These barriers motivate methodological approaches in this narrative review: system dynamics modeling, spatial analysis, and AI‐enhanced policy surveillance. Each approach is illustrated through a specific policy example. Recommendations for conducting policy‐relevant and methodologically rigorous research apply across all three approaches.

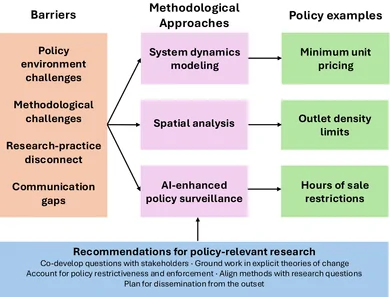

## 1 Introduction

Alcohol control policies, such as restrictions on availability and price increases through taxation, are among the most effective strategies for reducing alcohol consumption and its associated harms at the population level (Babor et al. 2022; Kilian et al. 2023; Nelson et al. 2013). Yet, in recent years, the United States has witnessed a steady relaxation of these policies (Babor et al. 2022; Nelson et al. 2015). This often takes the form of reduced enforcement at the local level, particularly for strategies targeting alcohol suppliers (Lenk et al. 2023; Nelson et al. 2015). Compounding this issue, many states and localities loosened alcohol regulations during the COVID‐19 pandemic; while some of these changes have been reversed or sunset, others remain in effect (Lemp et al. 2025; Peña et al. 2024). Given the immense public health burden of alcohol consumption in the United States (Matarazzo et al. 2025; Shield et al. 2024), and the strong evidence supporting these policies (Nelson et al. 2013), it is critical that we better bridge research and practice (Grummon et al. 2025).

Several barriers contribute to the gap between alcohol policy evidence and its use in policy decisions. Chief among these are methodological challenges, which include difficulties in measuring policy implementation and enforcement, accounting for local variation in analytic approaches that assess policy impacts, and evaluating long‐term effects. Researchers often prioritize questions that are less relevant to policymakers, and findings are rarely translated into actionable insights. Communication gaps, such as limited awareness of policy effectiveness and ineffective messaging, further undermine support, though the mechanisms differ. Public awareness campaigns may shift popular opinion, while trust‐building with individual policymakers and the cultivation of political will require different and more targeted strategies. Addressing these communication challenges requires complementary approaches, which we return to in our recommendations.

Scholarship around public policy distinguishes between enactment and impact (Emmons and Chambers 2021). Passing a law is only one step; it must be complemented by enforcement, evaluation, and revision to produce change (Baldwin et al. 2011). Meaningful policy‐relevant research requires a clear theoretical framework, codeveloped research questions, contextually specific data, rigorous methods that fit the research question, and effective dissemination of findings. Unfortunately, these ideals are inconsistently realized in alcohol policy research. Ways to strengthen the evidence base and its impact include refreshing our methodological approaches and collaborating more closely with stakeholders.

Strengthening the methodological rigor of alcohol policy research is not an end goal, but rather a precondition for generating evidence useful for both policymakers and the public. Rigorous methods produce findings that are credible, contextually specific, and actionable. These qualities can help research move from academic journals into the hands of decision‐makers. In this narrative review, we highlight three methodological approaches that can improve both the rigor and relevance of alcohol policy research: system dynamics modeling, spatial analysis, and AI tools for locating and coding policies. These three approaches were selected because they reflect methodological areas in which we have direct experience, and because each represents an underutilized but promising approach for alcohol policy research that has demonstrated value in adjacent fields. They are not intended as an exhaustive list of approaches that could strengthen alcohol policy research, but rather as illustrative examples that we believe have broad applicability to the field. For each approach, we illustrate its application using a specific policy example (minimum unit pricing, regulating alcohol outlet density, and limiting hours of sale in on‐premise outlets, respectively) and demonstrate how these methods can generate the kinds of actionable and relevant insights that policymakers need (see Figure 1). These approaches can help us move beyond estimating general associations toward generating specific, contextually grounded evidence capable of informing real policy decisions. We conclude by proposing guidelines for conducting research that is both methodologically rigorous and policy relevant—a precondition for rebuilding public and political will for effective alcohol control.

**FIGURE 1:**
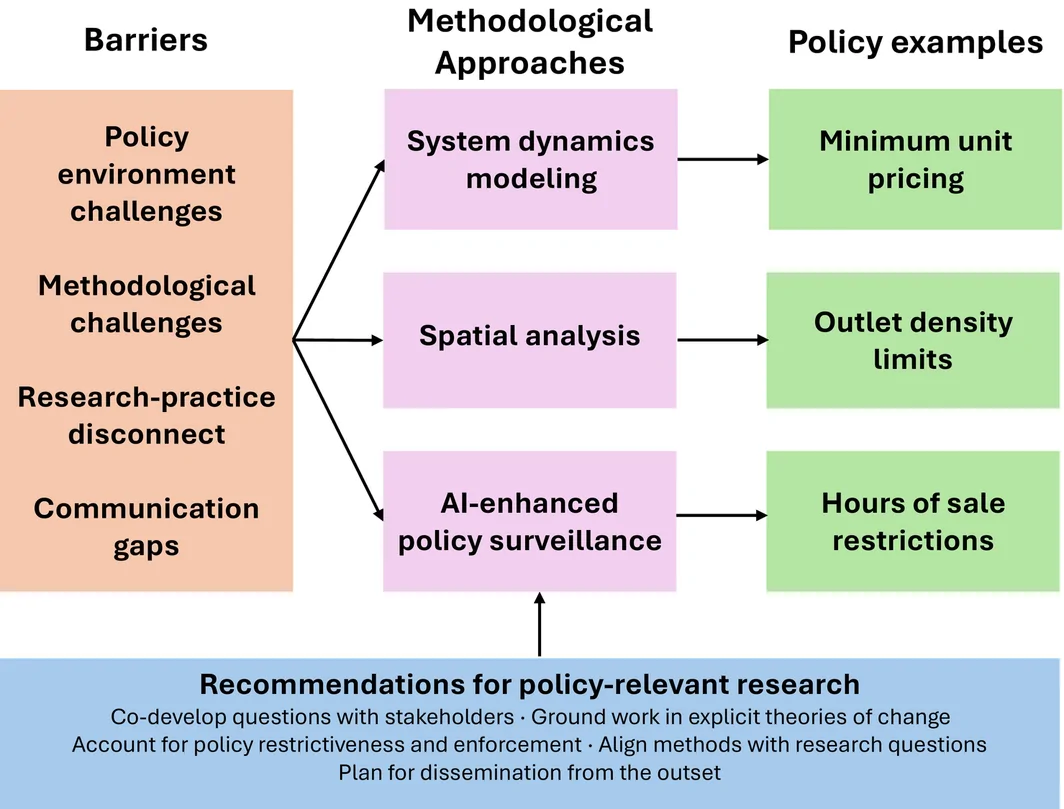
Connecting methods to policy: A framework for alcohol control research. Barriers to effective alcohol policy research (left) include the steady relaxation of alcohol control policies and reduced enforcement at the local level; methodological challenges in measurement, accounting for local variation, and evaluating long‐term effects; a research‐practice disconnect; and communication and awareness gaps. These barriers motivate three methodological approaches covered in this narrative review (center): System dynamics modeling, spatial analysis, and AI‐enhanced policy surveillance. Each approach is illustrated through a specific policy example (right): Minimum unit pricing, outlet density limits, and hours of sale restrictions, respectively. Recommendations for conducting policy‐relevant and methodologically rigorous research (bottom) apply across all three approaches.

## 2 Methodologic Approach: System Dynamics Modeling

System dynamics modeling is a simulation‐based systems science approach that represents systems as stocks (accumulations), flows (rates of change), and auxiliary variables (contextual factors) (Forrester 1961). Arranged into stock and flow diagrams, these elements and their relationships capture the high‐level balancing and reinforcing feedback loops, time delays, and nonlinear associations that define system behavior. System dynamics modeling emphasizes interactions between endogenous interventions and system components while allowing for the simultaneous evaluation of multiple policies under varying conditions (Sterman 2001). A range of participatory system dynamics approaches further support the inclusion of individuals with domain and lived expertise into the model development process, improving model relevance and credibility (Hovmand 2013). These attributes have led to the application of system dynamics modeling to support policy decision‐making and innovation across a wide range of domains (Homer and Hirsch 2006). For example, system dynamics approaches have been used to model the spread and control of infectious diseases, helping policymakers anticipate hospital demand and the effectiveness of containment measures during the COVID‐19 pandemic (Sun et al. 2023).

In alcohol policy research, system dynamics modeling has been used to elucidate mechanisms underlying drinking and driving among adolescents and young adults (Hosseinichimeh et al. 2022), develop models of multilevel factors that impact drinking events (Clapp et al. 2018), and map causes of alcohol‐involved sexual violence on college campuses (Moore et al. 2025). For example, Hosseinichimeh et al. qualitatively investigated drinking and driving among young adults, revealing key feedback loops contributing to impaired driving behaviors. These include a balancing loop linking DWI enforcement visibility to reductions in the number of alcohol‐impaired drivers (Hosseinichimeh et al. 2022). Quantifying these mechanisms in later work, the authors further identified impacts of potential policy levers (e.g., increased DWI enforcement, DWI restrictive laws, and alcohol taxes) and used system dynamics modeling to determine the combinations of multilevel policies most effective in reducing DWI‐related deaths (Hosseinichimeh et al. 2024, 2025). The CAMPUS study used a participatory approach to develop a qualitative stock and flow diagram of alcohol‐involved sexual violence on college campuses (Mair et al. 2025). Informed by the lived experiences of students and staff, the model identified mechanisms driving alcohol‐involved sexual violence and highlighted potential intervention targets. System dynamics modeling has also been used to model strategies for preventing risky drinking and alcohol use disorder onset, testing potential impacts of implementing primary, secondary, and tertiary prevention‐focused policies (Deutsch et al. 2023). These examples, among others (Apostolopoulos et al. 2018), highlight how system dynamics approaches can help uncover the policy‐relevant drivers of alcohol‐related harms while indicating promising opportunities for policy intervention (McGill et al. 2021).

### 2.1 Example Policy: Minimum Unit Pricing

Alcohol pricing policies, including excise taxes and minimum unit pricing, are some of the most cost‐effective strategies to reduce population‐level alcohol‐related harms (World Health Organization 2024). Minimum unit pricing policies establish a price floor per standard unit of alcohol as a financial deterrent against purchasing cheap, high‐strength beverages (Centers for Disease Control and Prevention 2024). Following early research demonstrating strong, consistent relationships between alcohol prices and consumption (Chaloupka et al. 2002; Wagenaar et al. 2009), minimum unit pricing gained prominence in the early 2000s, particularly in the United Kingdom, where rising alcohol‐related hospitalizations and mortality prompted policy intervention (Meier et al. 2010). Scotland became the first country to legislate minimum unit pricing in 2012, and after overcoming legal challenges (Katikireddi et al. 2014), implemented a minimum unit price of £0.50 per unit in 2018. Scotland's adoption attracted global attention, spurring similar policy adoptions and ongoing deliberations in several Canadian provinces (Thompson et al. 2017), Wales (Billan et al. 2025), and Australia (Lam et al. 2023; Taylor 2023), increasing recognition of minimum unit pricing as a targeted strategy for harm reduction among those who drink heavily.

To date, research on minimum unit pricing has largely focused on applying traditional econometric or epidemiologic modeling approaches to investigate policy impacts. Findings have highlighted the effectiveness of minimum unit pricing in reducing alcohol consumption (Kilian et al. 2023), alcohol‐related hospitalizations (Maharaj et al. 2023), and alcohol‐related mortality (Bertin et al. 2024; Boniface et al. 2017). By limiting access to cheap, high‐strength drinks, minimum unit pricing has been shown to reduce inequities in alcohol‐related harms (Clay et al. 2025) while minimizing impacts on alcohol retail revenue (Taylor et al. 2025).

While this growing body of work has provided a strong evidence base supporting the implementation of minimum unit pricing, several policy‐relevant questions persist. Given the recency of minimum unit pricing policies, there has been little investigation of potential long‐term impacts on chronic health outcomes (e.g., alcohol‐associated cancers, alcohol‐related liver disease) and economic adaptations to pricing changes (e.g., consumer substitution effects, industry responses). Open questions regarding the distributional effects of minimum unit pricing by age, income, and drinking patterns also remain, along with the underexplored potential for interactive effects between minimum unit pricing and other alcohol control policies.

### 2.2 Combining System Dynamics Modeling and Minimum Unit Pricing Research

System dynamics modeling is well suited to address outstanding questions about minimum unit pricing. Its capacity to capture feedback loops, time delays, and nonlinear relationships makes it particularly valuable for examining long‐term effects on chronic alcohol‐attributable diseases, consumer substitution behavior, and industry responses such as product reformulation to maintain competitiveness within new regulatory constraints. Importantly, given their focus on simultaneous policy evaluation, system dynamics models enable the analysis of potential synergistic or competing effects between minimum unit pricing and other alcohol control policies, such as excise taxation, alcohol outlet density regulations, and limits for on‐premise outlet hours of sale, providing insights into which combined interventions may yield additional benefits or diminishing returns.

To enhance the policy relevance of this work, researchers can draw on participatory system dynamics approaches to explicitly include policymakers, consumers most affected by alcohol pricing changes, and alcohol industry experts in the modeling process (Nelson et al. 2013). This can help ensure that models answer meaningful questions and capture important, policy‐relevant nuance. Collaborative engagement throughout the model development period can additionally foster trust in model assumptions and results while advancing a greater shared understanding of minimum unit pricing trade‐offs. Integrating system dynamics modeling with complementary systems science methods can leverage the strengths of each approach to more fully characterize minimum unit pricing impacts. Agent‐based modeling, for example, may better capture heterogeneity in consumers' individual‐level responses to changes in alcohol affordability, providing a helpful complement to the “big picture” system dynamics perspective (Boyd et al. 2024). By integrating empirical data from the extant literature, drawing on diverse collaborator perspectives, and incorporating actionable policy levers within a coherent systems framework, system dynamics modeling can advance the evidence base supporting future implementation of minimum unit pricing policies.

## 3 Methodologic Approach: Spatial Analysis

Spatial analyses combine methods from geography and statistics to examine spatial distributions of single variables and spatial relationships between multiple variables (Morrison et al. 2024). These methods are widely applicable for public health inquiry when one or more variables are a human health condition. Common steps include mapping individual variables, detecting areas with high concentrations (known as clusters), and relating variables to one another using multivariable statistical approaches such as regression (Kirby et al. 2017; Waller and Gotway 2004).

In alcohol research, spatial analytic methods have been used extensively for studies of retail alcohol outlets for more than three decades. This area of research is guided by availability theory, which proposes that alcohol consumption will rise in the population as availability increases (Babor et al. 2022), and the total consumption model, which suggests alcohol‐related harms are a function of population‐level consumption (Bruun 1975; Norström et al. 2005; Skog 1985). The earliest investigations found evidence to support associations among outlet densities, alcohol consumption, and alcohol‐related harms (Gorman and Speer 1997; Gruenewald et al. 1996; Scribner et al. 1994). Subsequent research has examined more nuanced aspects of these relationships (e.g., pairing specific types of outlets with specific harms), typically finding support for hypothesized associations (Popova et al. 2009). Based on this evidence, calls to limit densities of retail outlets are common (e.g., from the World Health Organization [World Health Organization 2024], Centers for Disease Control and Prevention [Centers for Disease Control and Prevention 2017], and Community Preventive Services Taskforce [Campbell et al. 2009]) and many city, county, state, and other regulatory authorities enact such policies to reduce alcohol‐related harm (Gruenewald et al. 1992).

Available research designs for investigating spatial relationships can be separated into two broad categories: individual‐level and ecological studies (Trangenstein et al. 2021). Individual‐level studies take individual people as the units of analysis (Sacks et al. 2020). The dependent variable is a measure of the individuals' alcohol consumption or experience of alcohol‐related harm, and the independent variable is a measure of their “exposure” to outlets. There are many ways to operationalize exposure in individual‐level studies (Morrison et al. 2019). The main approaches include point‐based measures, which geolocate individuals to precise locations (e.g., geocoded home address) then measure the distance to nearby outlets (Lu et al. 2018). Polygon‐based measures locate individuals within areas (e.g., ZIP code, city, county) and calculate exposure within the polygon to which they are joined. Point‐based measures provide greater spatial precision and flexibility, but polygon‐based measures are more commonly available, in part because they provide greater privacy for participants. Ecological studies offer a complementary perspective by comparing groups rather than individuals. In the case of spatial analyses of alcohol outlets, the groups are spatial polygons (Trangenstein et al. 2019), and the dependent measures are typically operationalized as counts of alcohol‐related harms (e.g., assaults [Gruenewald et al. 2022], drunk driver crashes [Morrison et al. 2016], child maltreatment reports [Freisthler et al. 2004]). The independent measures are outlet counts per population, per land area, or per roadway mile, depending on the guiding theory and available data (Sacks et al. 2020).

While evidence of the effects of outlets on alcohol use and alcohol‐related harm is widely available, there are important limitations. Most notably, the available data and methods do not support experimental study designs, so the best available evidence comes from observational studies and simulations (Castillo‐Carniglia et al. 2019). Confounding by area characteristics is also a major problem (Gmel et al. 2016). Environmental conditions tend to be highly spatially correlated, and alcohol outlets tend to locate in lower income and otherwise disadvantaged areas (Jin et al. 2018), where other conditions could be causally related to alcohol use and alcohol‐related harm.

### 3.1 Example Policy: Limiting Retail Alcohol Outlet Densities

Policies to limit retail alcohol densities have been used extensively as a tool to improve public health (Gruenewald et al. 1992). In the United States, the 18th Amendment essentially mandated a nationwide cap of zero licensed alcohol outlets from 1920 to 1933, before the 21st Amendment released that restriction. A wide range of density limits has since been enacted, amended, and sometimes repealed in cities, counties, and states. For instance, the California Business and Professions Code §23,816 (2022) limits the number of on‐premise licenses (e.g., bars, taverns) per county to less than one outlet per 2000 population. Similar limitations apply in other locations around the world. In urban areas of Western Australia, for example, large off‐premise outlets (> 400m^2^) must be located at least 5 km from any existing off‐premise outlet of comparable size.

Like the strategies to measure exposure to alcohol outlets, policies to limit outlet densities can also be operationalized using point‐based and polygon‐based approaches. Point‐based policies treat the location of individual outlets as the spatial anchor and place restrictions on the number of other licensed outlets that can be present within a distance buffer. By contrast, polygon‐based restrictions limit the number of licenses per geographic area, typically denominated by land area or population size.

As noted in the introduction, separating policy enactment from policy impact is critical. Limits on alcohol outlet density have multiple points of failure that can lead to a retail geography that does not reflect underlying policies. Judicial reviews (Cook et al. 2020), exceptions, grandfathering, and relocation of licenses across county boundaries all impede policy effectiveness. For example, even though California law limits the density of On Sale General licenses per county and per population (California Department of Alcoholic Beverage Control 2026a); comparing license counts (California Department of Alcoholic Beverage Control 2026b) with population estimates (California Department of Finance, Demographic Research Unit 2025), we calculate that a total of 32 of California's 58 counties have densities that exceed the legislated threshold.

### 3.2 Combining Spatial Analytic Models and Outlet Density Policies

When a city or county considers limiting the number of alcohol outlets, the decision requires a specific, locally grounded answer to the question *at what density do outlets begin to generate unacceptable levels of harm in this community*? Current research is poorly positioned to answer that question directly. Although research identifies overall associations between outlets and harms, many details germane to policy specification are lacking. Density caps imply a threshold above which outlet concentrations are unacceptable, yet most research assumes linear dose–response relationships—researchers should explicitly test for the nonlinearity these policies imply. Policies must necessarily distill complicated findings into simple messages, often to a single number, but research emphasizes measuring increasingly granular associations according to outlet types, harms, and contexts. Policies are written for individual jurisdictions with specific local conditions, yet research typically champions generalizable science, prioritizing findings that hold across many jurisdictions over those tailored to specific local contexts.

Spatial analytic tools are particularly well positioned to address these gaps directly, as they can be designed to incorporate local outlet data, population characteristics, and alcohol‐related outcomes, enabling jurisdictions to evaluate the likely impacts of specific density limits for their own context. Structuring analyses to directly inform policy wording and implementation, such as explicitly testing for nonlinear relationships and threshold effects rather than assuming linearity, and generating jurisdiction‐specific estimates rather than pooled associations, would help produce the kind of decision‐relevant evidence that policymakers can use. Aligning the design of spatial research with the specific decisions policymakers face is a concrete step toward bridging the gap between evidence and practice.

## 4 Method: Using AI Tools in Legal Epidemiology Research

The emerging field of legal epidemiology examines how laws, policies, and regulations shape patterns of disease, injury, and death, and provides guidance on how policies can promote health and safety in communities (Schnake‐Mahl et al. 2025). One important task in legal epidemiology is systematically identifying and measuring policies across a wide range of jurisdictions. In alcohol policy research, this means translating policy language into quantitative variables for analysis. This process is time‐consuming, resource‐intensive, and difficult to scale. AI tools can help researchers be more efficient when searching policy databases, extracting data, and coding policies following protocols developed for manually coding policies (Sim et al. 2023). Using AI tools may enable researchers to generate larger samples, examine differences across policy environments, and improve statistical power. If valid, these efficiencies could support larger studies and stronger evidence on the effectiveness of alcohol control policy and help identify more effective prevention strategies.

To our knowledge, AI tools have not been systematically evaluated to identify and code alcohol policies for analysis. However, researchers are beginning to use these tools in similar areas, which suggests they are potentially useful for alcohol policy research (Olawade et al. 2023). For example, a collaboration between the US Centers for Disease Control and Prevention and ICF International found that AI‐enhanced processes completed an analysis of COVID‐related policies and infectious disease outcomes in weeks, when manual methods were expected to take months (ICF n.d.). In alcohol epidemiology, AI tools have been used to analyze area‐level social media posts about alcohol consumption in relation to local alcohol outlet density (Ricard and Hassanpour 2021). AI‐enhanced methods have also searched electronic health records to identify cases of risky alcohol use, locating three times as many cases of risky alcohol use compared to standard diagnostic codes. This method could help determine whether mandatory screening and brief intervention policies are being followed in clinical practice (Vydiswaran et al. 2024).

The potential for AI tools to carry out policy surveillance and coding tasks is promising for future alcohol policy research and evaluation. The following section illustrates this potential through a specific example: using AI to examine local policies that restrict the hours of alcohol sales in on‐premise establishments.

### 4.1 Example Policy: Limiting Hours of Sale in On‐Premise Outlets

On‐premise hours of sale restrictions are legal limitations that specify the time periods when licensed establishments (e.g., restaurants, taverns, bars) may sell alcoholic beverages for consumption at their location, which may vary by day of the week, local ordinances, or license type. The goal of these policies is to prevent the sale of alcohol during times when alcohol‐related harms are most likely (Atkinson et al. 2018; Hahn et al. 2010), and these policies are relatively common in the United States. In our own prior work examining a national sample of 374 US municipalities, approximately half enacted such restrictions (Lenk et al. 2024; Nelson 2025).

### 4.2 Combining AI Tools and Hours of Sale Policy Analysis

Understanding how hours of sale policies vary across jurisdictions (and how that variation relates to alcohol‐related harms) requires researchers to first collect and measure policy restrictiveness across a range of jurisdictions. Our team developed manual search methods for locating and scoring hours of sale policies across multiple jurisdictions. However, we found this process to be time‐consuming and resource‐intensive. AI‐enhanced methods, if valid and reliable, have the potential to reduce the time and effort required to develop policy exposure variables. To explore the potential of AI‐enhanced methods, we compared an AI‐enhanced surveillance protocol to a rigorous manual protocol used previously to study local alcohol policies and alcohol‐related motor vehicle fatalities.

We adapted codes developed for state laws and sourced from the Alcohol Policy Information System (APIS) (Erickson et al. 2014) to create local policy codes to complement the state codes and allow us to consider both state and local policy simultaneously. We located local policies through searches of legal databases (e.g., Municode, American Legal Publishing, and eCode360) and municipal websites for the laws in effect as of January 1, 2019. Our manual search procedures used at least three researchers to independently locate and code policies in every jurisdiction. The key features of our hours of sale policy codes were the earliest permitted opening times, the latest allowable closing times, differences by day of the week, and any extended or restricted hours based on specific permit types. In our manual process, we examined any discrepancies across multiple coders and resolved them through discussion and consensus. The AI protocol included prompts to search specialized policy databases where we located policy language using our manual method, the same search terms we used for our manual process (e.g., Alcohol Chapter. Business Regulations, Zoning, days of sale, hours of sale, hours of operation), venue inclusion (e.g., bar, tavern, restaurant) and exclusions (e.g., private clubs, bottle clubs, golf courses, convention centers), and the same questions used to prompt our research staff conducting the manual coding. The answers to these questions were used to create a four‐level code for each jurisdiction ((i). No restrictions on hours of sale (except possibly on Sundays) have been enacted by the local government; (ii) latest closing time for establishments is 2:10 a.m. or later; (iii) latest closing time for establishments is earlier than 2:10 a.m. and there are provisions for later closing times, and (iv) latest closing time for establishments is earlier than 2:10 a.m. and there are no provisions for later closing times). We used three different AI tools (ChatGPT, Gemini, and Copilot) and used the same prompts with each platform to create a database for analysis. We selected a random set of 20 local jurisdictions where we had already completed manual coding for comparison. While the manual coding method is not a gold standard for judging validity, it does provide a means for assessing concurrent validity.

Our AI‐enhanced approach resulted in substantially reduced search time. We found that our manual searches and coding, including time for review and building consensus, required approximately 15–20 h of researcher time for each policy in each jurisdiction. In contrast, AI‐enhanced searches typically required less than 1 h per policy and jurisdiction. However, we observed low agreement levels between our manual methods and each of three widely available AI tools (ChatGPT = 40.0%; Gemini = 26.7%; Copilot = 20.0%) and similarly poor Kappa statistics (ChatGPT = 0.26; Gemini = 0.03; Copilot = −0.04). A review of the output from the coding suggests that AI tools were able to apply the decision rules provided to create numerical codes using identified policy language. However, despite specific prompts to search reliable databases for the legal language, the AI tools included other, less reliable, sources of information such as Reddit and Wikipedia. Problems such as poor data quality and hallucinations are well‐known challenges with using AI tools (Helmy et al. 2025) and may require additional effort to train existing platforms or dedicated tools for these specific purposes.

Our preliminary findings demonstrate the promise and limitations of AI‐enhanced policy coding. AI‐enhanced methods offered substantial improvements in the time required to find and code local policy language into quantitative codes for analysis. However, the poor agreement between AI‐enhanced methods and our manual methods indicates that AI tools are not yet valid and reliable substitutes. Improved speed and efficiency remain strong motivations for pursuing AI‐enhanced methods for policy coding. Potential paths for improving the reliability of AI‐enhanced methods include evaluating each stage of the coding process independently, optimizing prompts to use accurate source information for policies, and testing these across different jurisdictions, policies, and time frames to ensure they are consistent. Results from AI‐enhanced methods should be compared with manual methods at each step to identify places where the methods diverge. If the performance of AI tools can improve, they have the potential to transform the scale and efficiency of alcohol policy research.

## 5 Discussion: Recommendations for Generating Policy‐Relevant and Methodologically Rigorous Alcohol Research

The methodological approaches described in this narrative review each offer distinct pathways for strengthening the connection between research and policy action. System dynamics modeling, particularly when developed using participatory approaches, builds the kind of trust and shared understanding that is often missing from the research–policy relationship. When policymakers and community members are involved in constructing a model, they are more likely to find its assumptions credible and its outputs relevant to the decisions they face. This addresses a skepticism often found toward simulation‐based evidence that has limited its uptake for policy decision‐making. Spatial analysis offers a different but equally important contribution: the capacity to produce jurisdiction‐specific, decision‐relevant outputs. Rather than telling a policymaker that outlet density is generally associated with alcohol‐related harms, spatial methods can tell a specific jurisdiction what density threshold is associated with elevated harm in their community, given their local population, outlet landscape, and regulatory context. This is the type of concrete, actionable information required for policy decisions. AI‐enhanced policy surveillance addresses the mismatch between the pace of research and policy. Legislative windows open and close quickly, and evidence that arrives years after a policy debate has moved on has limited influence. By dramatically reducing the time required to characterize policy environments across jurisdictions, AI tools have the potential to bring research findings into alignment with real‐world policy timelines. The following recommendations build on these insights to provide a roadmap for producing research that is actionable, context‐sensitive, and capable of informing policy decisions at local, state, and federal levels.

1. Codevelop research questions with policymakers, practitioners, and community collaborators to ensure practical relevance. By building these partnerships early and collaborating throughout the research process, we can better identify pressing policy challenges and ensure that research outputs align with real‐world decision‐making needs. Incorporating lived experience and diverse perspectives can also improve the credibility and uptake of findings.
2. Base our studies on explicit theories of change that map policies to mechanisms and outcomes. Explicitly articulating causal pathways strengthens the interpretability of results and facilitates comparisons across studies and locations. This approach also supports the design of models such as system dynamics models that can test assumptions and identify leverage points for intervention.
3. Measure not only the presence or absence of a policy but also the degree of restrictiveness, implementation, and enforcement. Capturing these nuances allows researchers to explain heterogeneity in policy impacts and avoid misleading conclusions that treat policy presence or absence as equivalent to policy impact. For example, spatial analyses of outlet density policies should incorporate enforcement data to reflect actual retail geography rather than statutory limits.
4. Match methods to questions. Aligning analytic strategies with research objectives ensures methodological rigor and maximizes policy relevance. For example, we may want to use systems modeling for feedback and long‐term dynamics, spatial analysis for place‐based associations and spatial patterns, and AI enhanced policy surveillance and measurement for scalable policy coding. Further, combining approaches, such as integrating system dynamics with spatial data, can provide richer insights into how policies interact across geographic and systemic levels.
5. Prioritize planning for dissemination from the outset, considering how findings can be translated for non‐research audiences. Developing decision‐support tools, visualizations, and concise policy briefs can help bridge the gap between evidence and action. Researchers should anticipate threshold effects and local variation, balancing generalizable findings with context‐specific guidance for local use cases. Effective dissemination also requires researchers to develop communication strategies tailored to different audiences. Translating findings for policymakers means leading with decision‐relevant implications rather than methodological detail. We must provide a clear, actionable headline supported by accessible evidence, rather than leading with caveats and technical qualifications that obscure the practical message.

By integrating these recommendations into future research, we can build an evidence base that is both methodologically rigorous and actionable. Combining innovative analytic approaches with collaborator engagement and strategic dissemination can help translate evidence into policy action, ultimately building public support for effective alcohol control strategies to reduce alcohol‐related harms. At a time when alcohol‐related harms are rising and policy support is eroding, methodologically rigorous and accessible research is not merely an academic ideal, but a public health necessity.

## Funding

This study was supported by the National Institute on Alcohol Abuse and Alcoholism (NIAAA) under the grants R01AA029379 (PI: Mair), R01AA029112 (PI: Morrison), and R01AA026610 (PI: Nelson).

## Conflicts of Interest

The authors declare no conflicts of interest.

## Data Availability

Data sharing not applicable to this article as no datasets were generated or analysed during the current study.
